# Improved Global Surface Temperature Simulation using Stratospheric Ozone Forcing with More Accurate Variability

**DOI:** 10.1038/s41598-018-32656-z

**Published:** 2018-09-27

**Authors:** Fei Xie, Jianping Li, Cheng Sun, Ruiqiang Ding, Nan Xing, Yun Yang, Xin Zhou, Xuan Ma

**Affiliations:** 1 College of Global Change and Earth System Science, Beijing Normal University, Beijing, China; 2 Laboratory for Regional Oceanography and Numerical Modeling, Qingdao National Laboratory for Marine Science and Technology, Qingdao, China; 3 State Key Laboratory of Numerical Modeling for Atmospheric Sciences and Geophysical Fluid Dynamics, Institute of Atmospheric Physics, Chinese Academy of Sciences, Beijing, China; 4 Beijing Meteorological Observatory, Beijing, China; 5 Plateau Atmosphere and Environment Key Laboratory of Sichuan Province, College of Atmospheric Science, Chengdu University of Information Technology, Chengdu, China

## Abstract

Increasingly, studies have pointed out that variations of stratospheric ozone significantly influence climate change in the Northern and Southern hemispheres. This leads us to consider whether making the variations of stratospheric ozone in a climate model closer to real ozone changes would improve the simulation of global climate change. It is found that replacing the original specified stratospheric ozone forcing with more accurate stratospheric ozone variations improves the simulated variations of surface temperature in a climate model. The improved stratospheric ozone variations in the Northern Hemisphere lead to better simulation of variations in Northern Hemisphere circulation. As a result, the simulated variabilities of surface temperature in the middle of the Eurasian continent and in lower latitudes are improved. In the Southern Hemisphere, improvements in surface temperature variations that result from improved stratospheric ozone variations influence the simulation of westerly winds. The simulations also suggest that the decreasing trend of stratospheric ozone may have enhanced the warming trend at high latitudes in the second half of the 20th century. Our results not only reinforce the importance of accurately simulating the stratospheric ozone but also imply the need for including fully coupled stratospheric dynamical–radiative–chemical processes in climate models to predict future climate changes.

## Introduction

Stratospheric ozone protects life on Earth by absorbing ultraviolet radiation^1–3^. In polar regions, stratospheric ozone depletion results in a temperature decrease in the stratosphere through a strong radiative cooling effect, which enhances the meridional gradient of temperature and westerly winds^4–7^. The process eventually leads to a stronger polar vortex, which has a significant influence on the tropospheric climate in both hemispheres^8–15^. This phenomenon is much stronger in the Antarctic than in the Arctic.

In recent years, the impacts of stratospheric ozone on climate changes have received widespread attention. The connection between Arctic stratospheric ozone (ASO) variations and tropospheric climate change over the Northern Hemisphere has been revealed in observations and simulations. As early as the 1990s, some studies noted a significant surface temperature warming trend in the mid-to-high latitudes of the Eurasian continent since the late 1970s^16–18^. Though inevitably connected to the increase in atmospheric greenhouse gas concentrations, the warming trend was also found to be strongly associated with enhanced westerly winds caused by ASO depletion^19,20^. A large fraction of the variability in the March–April surface temperature in certain regions of the Northern Hemisphere is associated with variations in March Arctic ozone from observations^21^. Recently, numerous modeling studies have analyzed the possible linkage between ASO and tropospheric climate^22–24^. Previous studies found that the signal of spring ASO changes can propagate to the ground, resulting in changes to the climate of the mid-to-high latitudes of the Northern Hemisphere; this includes changes to sea level pressure (SLP) and the tropospheric jet^21,25^. Based on observations and simulations, it is found that the northern stratospheric circulation anomalies induced by ASO radiative anomalies could cause North Pacific SST anomalies (Victoria Mode anomalies)^24,25^. The SST anomalies link to the North Pacific circulation that in turn influences El Niño–Southern Oscillation (ENSO)^24^ and tropical rainfall^26^.

Due to substantial emissions of ozone-depleting substances, Antarctic stratospheric ozone losses exceeded 40% of the total ozone at the end of the 20th century^27–31^. The Antarctic ozone hole has been shown to have a significant influence on the Southern Hemispheric climate^32–39^. Some studies pointed out that the signal of enhanced winds related to the Southern Annular Mode can extend from the stratosphere to the surface^37,40^, causing climate warming on the eastern Antarctic Peninsula and cooling in the Antarctic interior during recent decades^41,42^, although other studies suggesting that the warming and cooling trends may be a manifestation of natural variability^43,44^. Previous studies demonstrated that stratospheric cooling caused by the Antarctic ozone hole resulted in a poleward shift of the extratropical westerly jet and extension of the Hadley cell in the Southern Hemisphere during austral summer^40,45^. Furthermore, the displacement of the westerly jet was also associated with a poleward shift of the subtropical dry zone, with increased rainfall in mid latitudes and reduced rainfall in the high latitudes of the Southern Hemisphere^34,46^. In addition, it has been shown that the variations in storm tracks and ocean circulation in the Southern Hemisphere were clearly affected by the Antarctic ozone hole^47–49^.

Previous studies showed that the variations in stratospheric ozone in the northern and southern high latitudes could significantly influence climate changes of Northern and Southern hemispheres. This inspires us to check whether the ozone forcing in our climate model is in agreement with observations. If there is a significant difference between the ozone forcing and the observed ozone variations, it is likely to have a negative impact on the quality of the simulation results.

**Figure 1:**
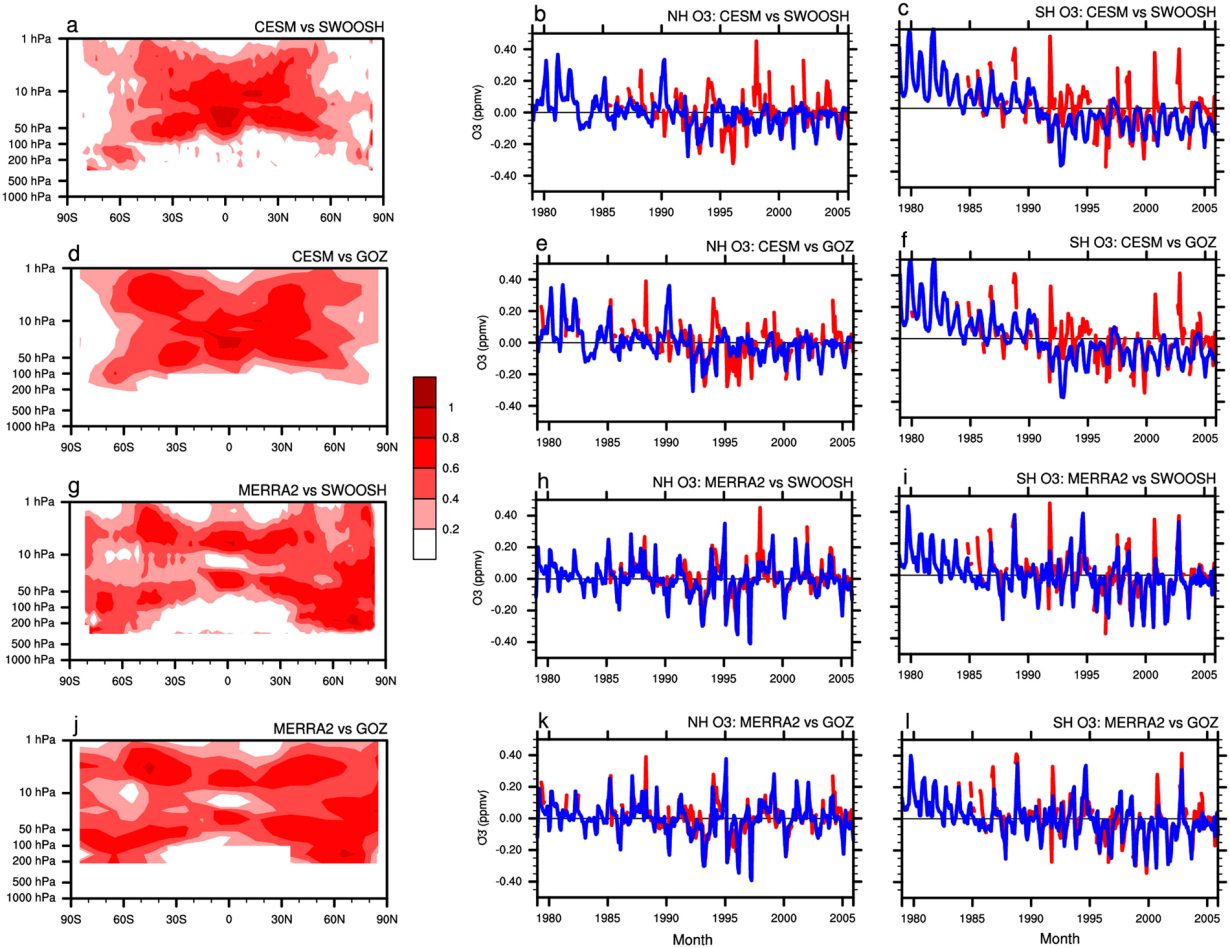
Comparison of forcing ozone and observed ozone. (**a**) Correlation coefficients between stratospheric ozone variations in the WACCM4 model input data and in SWOOSH for the period 1979–2005. Input data is the standard CESM ozone forcing used in WACCM4. (**b**) Variations in stratospheric ozone averaged over the region 60–90°N and 150–50 hPa from WACCM4 model input data (blue line) and (**c**) over the region 60–90°S and 200–100 hPa from SWOOSH (red line). (**d**–**f**) Same as (**a**–**c**), but for stratospheric ozone from WACCM4 model input data (blue line) and GOZCARDS (red line). (**g**–**i**) Same as (**a**–**c**), but for stratospheric ozone from MERRA2 (blue line) and SWOOSH (red line). (**j**–**l**) Same as (**a**–**c**), but for stratospheric ozone from MERRA2 (blue line) and from GOZCARDS (red line).

Here, we investigate the stratospheric ozone forcing in a fully coupled global climate model, the Community Earth System Model (CESM) Whole Atmosphere Community Climate Model, version 4 (WACCM4) with WACCM4-GHG scheme (more details of the model are given in section Simulations and Data), and compare the ozone variations specified in the model with observations. Figure 1a shows the correlation coefficients between the stratospheric ozone variations from WACCM4 model forcing (based on CMIP5 ozone output) and Stratospheric Water and OzOne Satellite Homogenized (SWOOSH) (see the data description in section Simulations and Data). It is found that the stratospheric ozone variations in the WACCM4, which is based on CMIP5 ozone output, are well correlated with the observations in mid and lower latitudes, but not in the high latitudes of the Northern and Southern hemispheres. It is because ozone in the polar vortex regions has much higher variability than lower latitudes which might require much larger ensemble size to capture the observed ozone variations^50^. Figure 1b and c shows the variations in stratospheric ozone averaged over the region 60°–90°N and 150–30 hPa and in the region 60°–90°S and 200–50 hPa for the period 1979 to 2015, respectively. The both variations in ozone from WACCM4 forcing data (based on CMIP5 ozone output) and from observations (SWOOSH) showed a clear decreasing trend from 1979 to 2005 in the stratosphere at the two poles. However, the ozone variability in WACCM4 is evidently different from that in observations in the two regions. These results can be further confirmed by observations from Global Ozone Chemistry and Related trace gas Data Records for the Stratosphere (GOZCARDS) (see section Simulations and Data, data description) (Fig. 1d–f). Note that in these two regions the variability and depletion of ozone concentration are most pronounced^51^ and have the most important influence on surface climate in the Northern and Southern hemispheres^21–23,25,34–36,52^.

Figure 1a–f shows that although the trend of stratospheric ozone is well represented in the WACCM4, the stratospheric ozone variability from the WACCM4 is not in good agreement with the observed stratospheric ozone variability in the high latitudes. This poor agreement is also found in many other climate models^53,54^. It is well known that a large number of model evaluation studies showed that although the simulated spatial patterns of climatic elements in historical experiments from Coupled Model Intercomparison Project 3 (CMIP 3) to CMIP 5 are closer to the observations, the simulated variability of these climatic elements needs further improvement. Here, we pose a question: if we replace the ozone forcing in WACCM4 with ozone data that have variations closer to those observed, would this help to improve the simulated climatic elements in the reproduced experiments by WACCM4?

Figure 1g shows the correlation coefficients between stratospheric ozone variations from MERRA2 and SWOOSH (see section Simulations and Data of the data description). It is found that the stratospheric ozone variations from MERRA2 not only have a good correlation with those from SWOOSH in the mid and lower latitudes but also in the high latitudes in both hemispheres. Figure 1h,i shows the variations in stratospheric ozone averaged over the region 60°–90°N and 150–30 hPa and over the region 60°–90°S and 200–50 hPa, respectively. The ozone variability based on MERRA2 is in good agreement with that based on SWOOSH over these two regions. These results can be further confirmed by the observations from GOZCARDS (Fig. 1j–l) (see section Simulations and Data). Replacing the originally specified stratospheric ozone forcing in WACCM4 by MERRA2 ozone to perform the historical experiment would help to test the sensitivity of the simulated climate to stratospheric ozone forcing. Note that MERRA2 ozone data are used because they have no missing values in the stratosphere compared with ozone from SWOOSH and GOZCARDS. The global surface temperature variability partly reflects the characteristics of global climate change. In this study, we choose the surface temperature to check whether improving the stratospheric ozone variability improves the simulation of global climate change in WACCM4. The experimental design is described in the next section.

## Results

**Figure 2:**
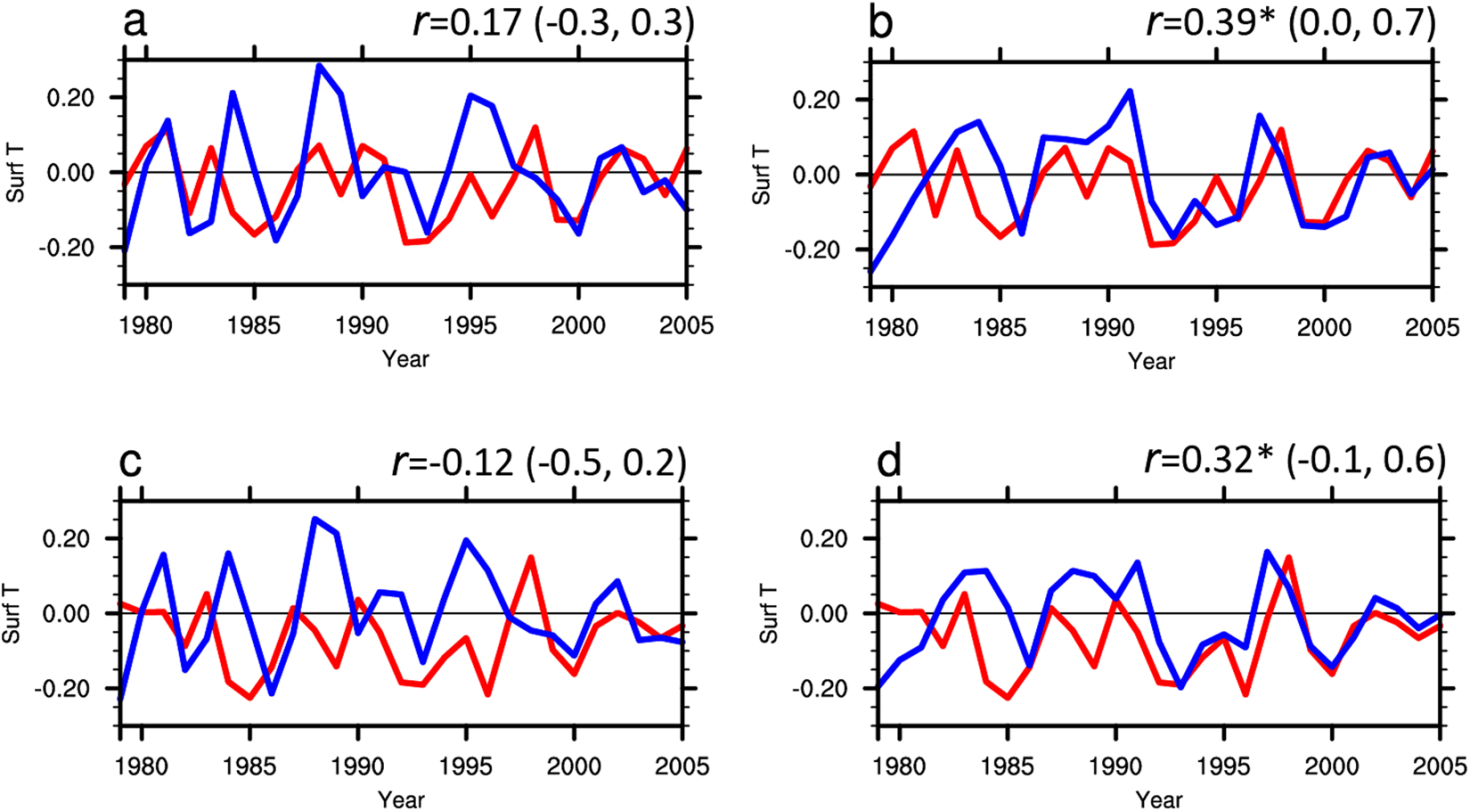
The time series of simulated globally averaged surface temperature. (**a**) Time series of globally averaged annual surface temperature anomalies for the period 1979–2005 from E_1–3_ (blue line) and GISTEMP (red line). (**b**) Same as (**a**), but for surface temperature from E_4–6_ (blue line) and GISTEMP (red line). (**c**) Same as (**a**), but for surface temperature from E_1–3_ (blue line) and HadCRUT4 (red line). (**d**) Same as (**a**), but for surface temperature from E_4–6_ (blue line) and HadCRUT4 (red line). The anomalies are obtained by removing the seasonal cycle and the time series are detrended. The correlation coefficients (*r*) between the two lines are shown in the top right corner and the “*” means *r* is significant at the 95% confidence level. The values in bracket are uncertainty range for the correlation coefficient at the 95% confidence level.

We first compare the simulated interannual variability of global average surface temperatures from E_1–3_ and from E_4–6_ with the observations. Taking the surface temperature from GISTEMP as the observations, Fig. 2a,b shows the time series of global average surface temperature for the period 1979–2005 from simulations and observation. The correlation coefficient between global average surface temperature from E_1–3_ and from GISTEMP is insignificant (*r* = 0.17, Fig. 2a), while the correlation coefficient between surface temperature from E_4–6_ and from GISTEMP is significant (*r =* 0.39, Fig. 2b). This result can be further confirmed by surface temperature from HadCRUT4 (Fig. 2c,d). This illustrates that the simulated variability of global average surface temperature is improved when the ozone forcing in the model is replaced by MERRA 2 ozone.

**Figure 3:**
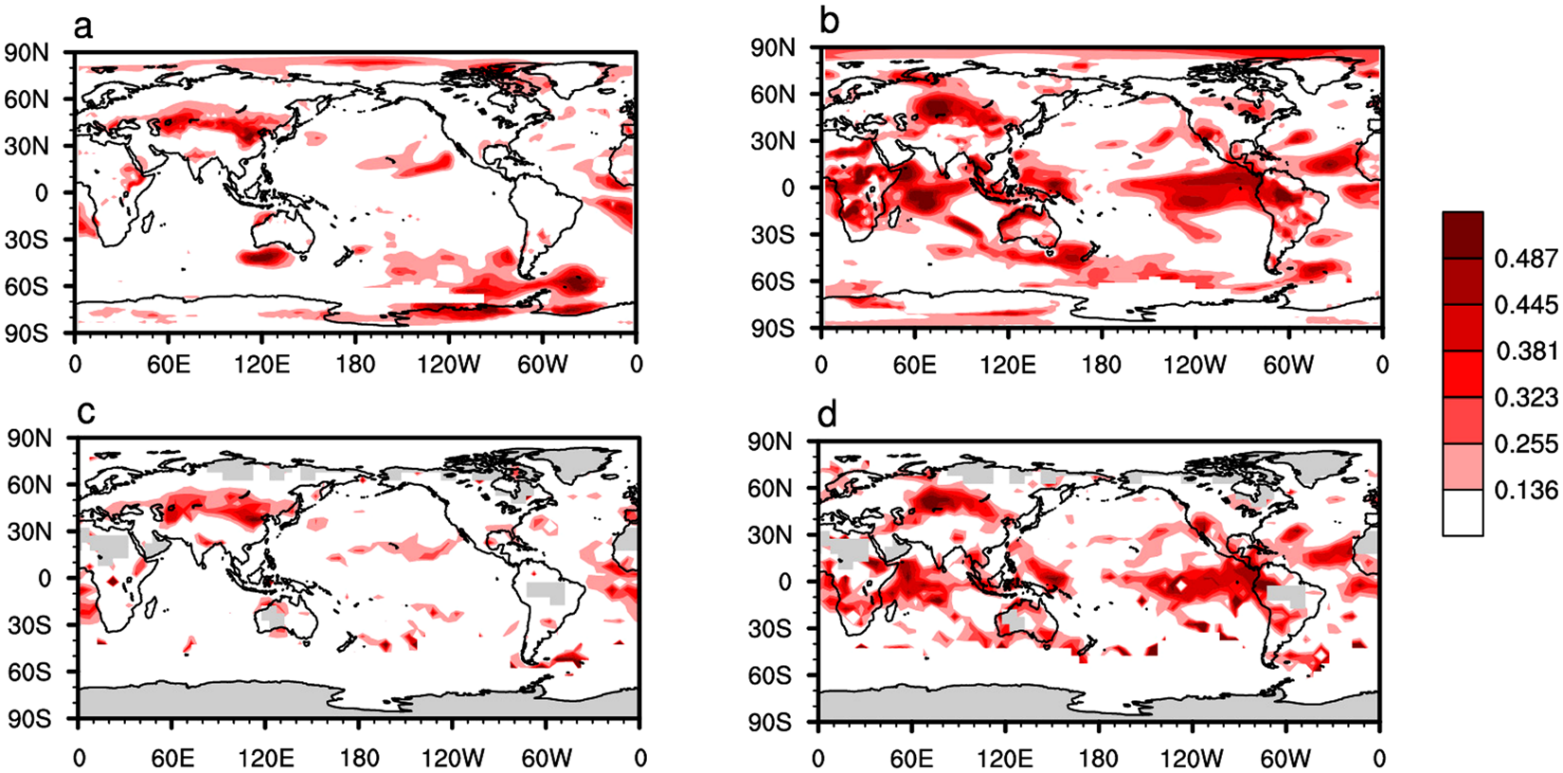
The spatial distribution of simulated global surface temperature. (**a**) Horizontal distribution of correlation coefficients between global surface temperature variations from E_1–3_ and GISTEMP for the period 1979–2005. (**b**) Same as (**a**), but for surface temperature from E_4–6_ and GISTEMP. (**c**) Same as (**a**), but for surface temperature from E_1–3_ and HadCRUT4. (**d**) Same as (**a**), but for surface temperature from E_4–6_ and HadCRUT4. Surface temperature variations are obtained by removing the seasonal cycle and are detrended. Only positive correlations coefficients are shown. In the color bar, the values 0.136, 0.255, 0.323, 0.381, 0.445 and 0.487 correspond to the 80%, 85%, 90%, 95%, 99% and 99.9% confidence levels, respectively.

Figure 3a,c shows the horizontal distributions of correlation coefficients between simulated (E_1–3_) and observed (GISTEMP/HadCRUT4) global surface temperature. One most obvious feature in the two panels is that the correlation coefficients in the middle of the Eurasian continent are significant (Fig. 3a,c), meaning that there is a certain degree of similarity between simulated surface temperature variations from E_1–3_ and the observations in the middle of the Eurasian continent. However, the correlation coefficients in other places are almost insignificant. It illustrates that the modeling capability of the climate model to surface temperature variability needs to be further improved.

Figure 3b,d shows that the simulated surface temperature variations from E_4–6_ are significantly correlated with observations (GISTEMP/HadCRUT4) in many regions. The two main features are: first, in the middle of the Eurasian continent the correlation coefficients between surface temperatures from E_4–6_ and from observations (Fig. 3b,d) are much larger than those between surface temperatures from E_1–3_ and from observations (Fig. 3a,c). Second, in the lower latitudes the correlation coefficients are significantly improved in E_4–6_ compared with those in E_1–3_. It means that the improved stratospheric ozone forcing in E_4–6_ compared with in E_1–3_ mainly improves simulated surface temperature in the middle of the Eurasian continent and in lower latitudes, which is responsible for the improved simulation of global average surface temperature in E_4–6_ compared with that in E_1–3_ (Fig. 2). In the following paragraphs we will discuss the mechanisms by which improved ozone forcing improves the surface temperature simulation over these two major areas.

Figure S1a,b shows the distributions of correlation coefficients between observed surface temperature and ASO. The variations in ASO are closely linked to surface temperature changes in the middle of the Eurasian continent. This phenomenon has been reported in previous studies using observations^16–18,21^ and simulations^22,25^. It illustrates that the ASO variations connected with the surface temperature anomalies over the middle of the Eurasian continent can be found in observations, supporting that simulated surface temperature in the middle of the Eurasian continent is improved in E_4–6_ as a result of improved stratospheric ozone forcing.

Previous studies showed that the surface temperature anomalies were strongly connected with westerly wind variations over the middle of the Eurasian continent caused by ASO changes^19,20^. In order to understand how improving ozone forcing would improve the surface temperature simulation over the middle of the Eurasian continent, we have to first build a bridge between the circulation anomalies in the middle of the Eurasian continent and in the northern high-latitude stratosphere.

Several studies have presented evidences suggesting that variability in the stratospheric polar vortex has a substantial impact on the circulation of the troposphere^8,55,56^ by the downward control principle^57^ or by tropospheric eddy momentum feedback^13,58,59^. Thus, a possible pathway for northern high-latitude stratospheric circulation anomalies to affect circulation anomalies over the middle of Eurasian continent may involve two steps: a high-latitude stratosphere-to-troposphere vertical pathway and an Arctic-to-Eurasia horizontal teleconnection. The high-latitude stratosphere-to-troposphere vertical pathway has been investigated^25^. They pointed out that the region 60°–90°N, 180°–120°W is the possible location of the main tunnel through which Arctic stratospheric circulation anomalies caused by the ASO changes reach down into the troposphere. To study the horizontal propagation of circulation anomalies from the Arctic to Eurasia in detail, the ray paths of waves at 200 hPa generated by the perturbed circulation over the region 60°–90°N, 180°–120°W are shown in Fig. S2. The wave ray paths represent the climate teleconnections; i.e., the propagation of stationary waves in realistic flows. The calculation of the wave ray paths and application of the barotropic model are described in detail by previous studies^60,61^. We found that the Rossby waves generated by the perturbed circulation over the north polar upper troposphere mainly propagate eastward along a line of latitude; after almost circling the earth, the Rossby waves reach the middle of the Eurasian continent in about 15 days. The above analysis establishes a connection between the northern high-latitude stratospheric circulation anomalies and circulation variations over the middle of the Eurasian continent. This may be why previous studies found that westerly wind variations over the middle of the Eurasian continent were influenced by ASO changes^19,20^.

Figure 1a,d,g,j shows that the ozone forcing in E_4–6_ is mainly improved in the high-latitude stratosphere of both hemispheres. This implies that the improved surface temperature simulation is mainly related to the improved high-latitude stratospheric ozone variability. The variations in ASO first influence Arctic polar stratosphere temperature by radiative processes. It is well known that a cooler (warmer) polar stratosphere strengthens (weakens) the temperature gradient from the tropics to the Pole. According to the thermal wind relationship, this situation results in a stronger (weaker) stratospheric polar vortex. Thus, improving the high-latitude stratospheric ozone variability should first improve the simulated stratospheric temperature and circulation at high latitudes. We compare the simulated stratospheric temperature and circulation of the Northern Hemisphere high latitudes from E_1–3_ and from E_4–6_ with observations. Taking the temperature and zonal wind from ERA-Interim for the period 1979–2005 as the references, the correlation coefficients between stratospheric temperature from E_4–6_ and from ERA-Interim are larger than the correlation coefficients between stratospheric temperature from E_1–3_ and from ERA-Interim (Fig. S3a). The improved simulations of stratospheric temperature variability over the North Pole improve the simulation of stratospheric circulation variability in E_4–6_ (Fig. S3b). According to the above established connection between the northern high-latitude stratospheric circulation anomalies and circulation variations over the middle of the Eurasian continent, the improved simulation of Arctic stratospheric circulation variability in E_4–6_ (Fig. S3b) explains how improved ozone forcing improves the surface temperature simulation over the middle of the Eurasian continent.

**Figure 4:**
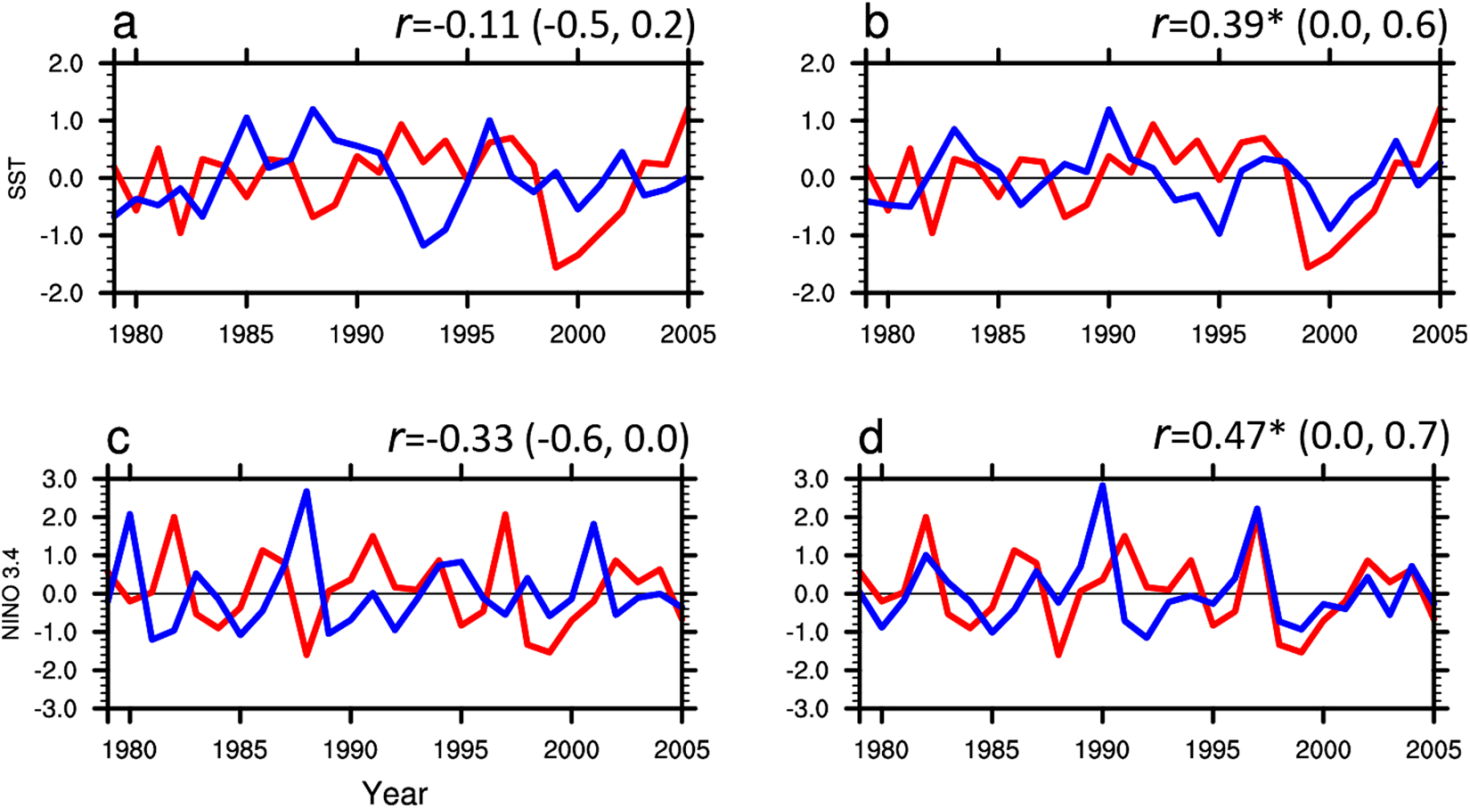
The time series of simulated VM and ENSO indices. (**a**) Time series of spring Victoria Mode index for the period 1979–2005 from E_1–3_ (blue line) and HadSST (red line). (**b**) Same as (**a**), but for spring Victoria Mode index from E_4–6_ (blue line) and HadSST (red line). (**c**) Time series of winter ENSO index for the period 1979–2005 from E_1–3_ (blue line) and from the Climate Prediction Center/NOAA (red line). (**d**) Same as (**a**), but for the winter ENSO index from E_4–6_ (blue line) and from the Climate Prediction Center/NOAA (red line). The correlation coefficients (*r*) between the two lines are shown in the top right corner and the “*” means *r* is significant at 95% confidence level. The values in bracket are uncertainty range for the correlation coefficient at the 95% confidence level.

Why is the simulated tropical surface temperature significantly improved in E_4–6_ compared with E_1–3_? A possible connection is established from the ASO to the tropical surface temperature^23^. They found that the ASO radiative anomalies influence the Victoria Mode^62,63^, which links to the North Pacific circulation that in turn influences ENSO. Figure 4 compares the simulated Victoria Mode and ENSO variations from E_1–3_ and from E_4–6_ with observations. Taking the Victoria Mode variations from HadSST and ENSO index from Climate Prediction Center/NOAA for the period 1979–2005 as the references, the correlation coefficients between the Victoria Mode (ENSO) from E_4–6_ and from HadSST (Fig. 4b,d) are larger than those between the Victoria Mode (ENSO) from E_1–3_ and from HadSST (Fig. 4a,c). Since the simulation of ENSO variations is improved as a result of improved ASO variations, Fig. 3b,d shows that the pattern of improved surface temperature over the eastern Pacific is similar to the pattern of ENSO. It is well known that ENSO links the SST change over the Indian Ocean and Indo Pacific warm pool via the Walker circulation. Thus, the simulations of surface temperature over the Indian Ocean and Indo Pacific warm pool are also improved in E_4–6_ (Fig. 3b,d) compared with E_1–3_ (Fig. 3a,c)_._

Note that the simulations of surface temperature variability in parts of the Southern Hemisphere are improved in E_4–6_ (Fig. 3b,d) compared with E_1–3_ (Fig. 3a,c), in particular in the middle latitude of the Southern Hemisphere. Previous studies demonstrated that stratospheric cooling caused by the Antarctic ozone hole results in a poleward shift of the extratropical westerly jet and extension of the Hadley cell in the Southern Hemisphere. Furthermore, the displacement of the westerly jet is also associated with a poleward shift of the subtropical dry zone, with increased rainfall in mid and lower latitudes of the Southern Hemisphere^34–36,40,45,46^. The specified ozone forcing improvement in E_4–6_ in the southern high-latitude stratosphere (Fig. 1a,d,g,j) improves the simulations of stratosphere temperature in southern high latitudes (Fig. S4a) and the westerly jet in the southern middle and lower latitudes in E_4–6_ (Fig. S4b) compared with E_1–3_. The improved simulation of the Southern Hemisphere westerly jet helps to explain the improved simulation of surface temperature variability in the Southern Hemisphere (Fig. 3b,d).

## Conclusions and Discussion

Previous studies have shown that the variations in stratospheric ozone in the northern and southern high latitudes could significantly influence changes in the Northern and Southern Hemisphere climate. However, the stratospheric ozone variations specified in many climate models are not in good agreement with the observed stratospheric ozone variations. This led us to consider whether part of the deviation between the simulated and observed variabilities of climatic elements from a large number of models is related to the poor agreement between observed ozone variations and the stratospheric ozone forcing applied in the climate models. Here, we investigated the improvement of simulated surface temperature by replacing the original specified ozone forcing with MERRA2 ozone in WACCM4.

Replacing the original specified stratospheric ozone forcing with MERRA2 stratospheric ozone improved the variability of global average surface temperature simulated by WACCM4. The main regions where the simulated surface temperature is significantly improved are the middle of the Eurasian continent and at lower latitudes. The improved stratospheric ozone variations improve the simulated variability of stratospheric temperature and circulation in both hemispheres at high latitudes. In the Northern Hemisphere, the ASO variations first influence the circulation over the north polar upper troposphere. The Rossby waves generated by the disturbed circulation over the north polar upper troposphere propagate eastward along a line of latitude and then reach the middle of the Eurasian continent to influence the local circulation and surface temperature. We found that the improved stratospheric ozone forcing improves the simulated variations of the Victoria Mode, which improves the simulation of ENSO variations and surface temperature changes over the Indian Ocean and Indo Pacific warm pool. In the Southern Hemisphere, improved stratospheric circulation variations resulting from improved Southern Hemisphere stratospheric ozone variations influence the simulation of westerly winds. This helps to improve the simulation of surface temperature in the Southern Hemisphere.

**Figure 5:**
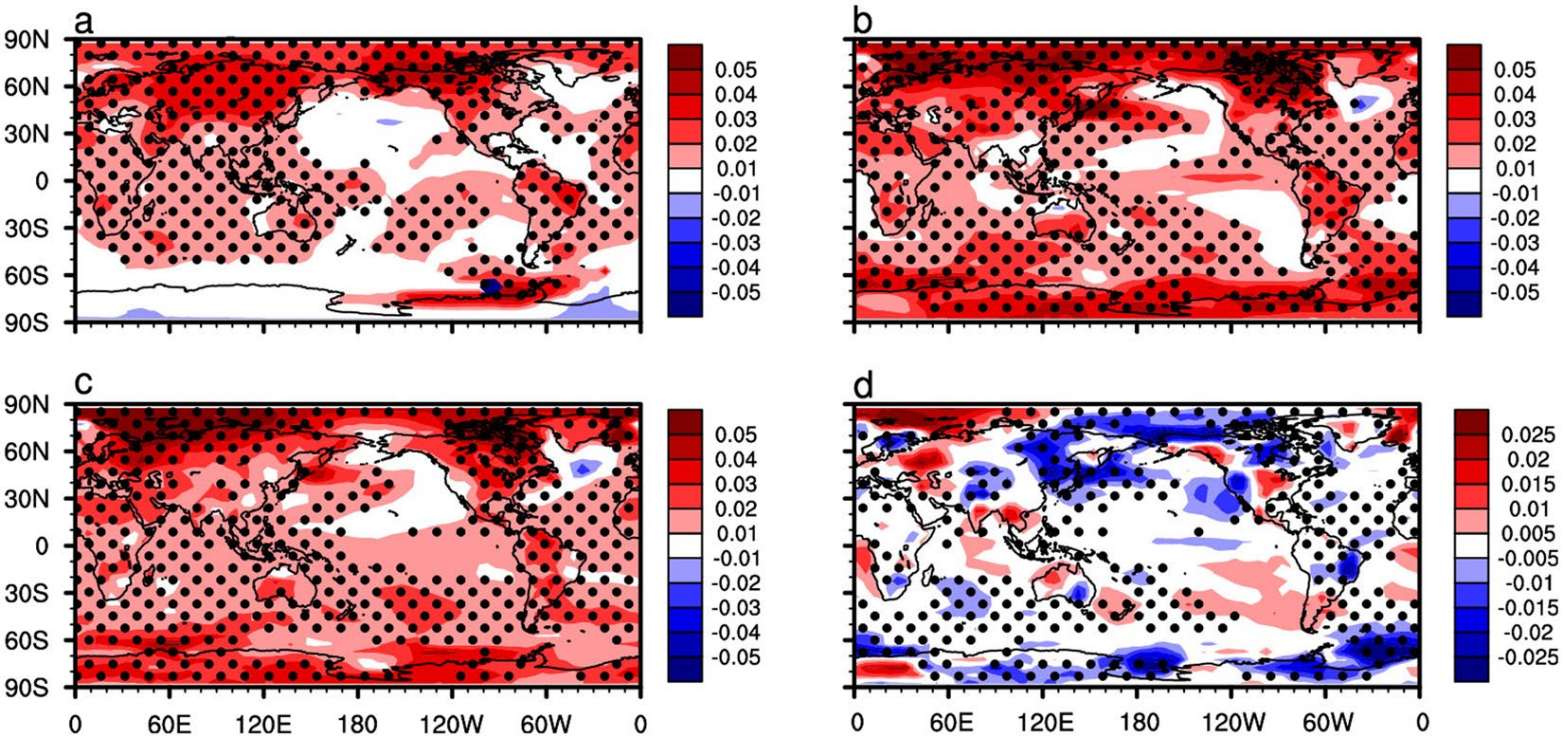
The simulated trend of global surface temperature. (**a**) Linear trend (K/year) of global surface temperature for the period 1955–2005 from GISTEMP. (**b**,**c**) are the same as (**a**), but from E7 and E8, respectively. (**d**) The difference between (**c**,**b**). Dots denote significance at the 99% confidence level, according to Mann-Kendall trend significance test.

This study focuses on the influence of stratospheric ozone variability on surface temperature variability. Here, we briefly discuss the influence of stratospheric ozone trend on surface temperature trend. Two additional experiments (E_7_ and E_8_) are performed. E_7_ is a historical simulation covering the period 1955–2005. E_8_ is the same as E_7_, but the linear trend of specified ozone forcing in the stratosphere was removed. An overview of the experiments (E_7_ and E_8_) is given in Table 1. The influence of stratospheric ozone trend on surface temperature can be obtained by comparing the results of E_7_ and E_8_. Figure 5a,b shows the linear trends of global surface temperature for the period 1955–2005 from GISTEMP and E_7_. In general, WACCM4 simulated the global warming trend fairly well compared with the observations, except that the simulated linear trend at high latitudes in northern Eurasia is evidently larger than that observed and there is a warming trend over the North Pacific in the simulation that is not seen in the observations. Figure 5c shows the linear trends of global surface temperature from E_8_. The linear trends in E_8_ are weaker than those in E_7_ in both hemisphere high latitudes. This feature is easier to see in Fig. 5d, which shows the difference of linear trends between E_8_ and E_7_. It is well known that stratospheric ozone decreased from 1955 to 2005. Figure 5d implies that the decreasing trend of stratospheric ozone may have enhanced the warming trend in both hemisphere high latitudes during the second half of the 20th century. Figure S5 shows the linear trend in surface temperature for the period 1955–1995. It shows that the warming trends in the Arctic and Antarctic from 1955 to 1995 (Fig. S5) are larger than those from 1955 to 2005 (Fig. 5b). This means that the behavior of trend variations in surface temperature during 1955–2005 is similar to those for ozone. The possible reasons why decreased stratospheric ozone warms high latitudes, and whether the Arctic stratospheric ozone decrease would contribute to the Arctic amplification effect, are open questions that deserve further investigation. Recently, a study found that Arctic stratospheric polar vortex possibly contributed to and sustained the recent hiatus in Eurasian winter warming^64^. Whether the stratospheric recovery after 2000 would also contribute to the hiatus of global warming is also an interesting question.

**Table 1:** Fully coupled CESM–WACCM4 experiments with various specified ozone forcings (WACCM-GHG scheme).

Experiments	Specified ozone forcings
E_1_ E_2_ E_3_	E_1_ is a historical simulation covering the period 1979–2005. Transient run using case B_1955–2005_WACCM_SC_CN in CESM. All natural and anthropogenic external forcings for E_1_ based on original CESM input data. Note that the specified ozone forcing for 1979–2005 was derived from the CMIP5 ensemble mean ozone output. The specified ozone forcing was named ghg_forcing_1955–2005_CMIP5_EnsMean.c140414.nc, and can be downloaded at https://svn-ccsm-inputdata.cgd.ucar.edu/trunk/inputdata/atm/waccm/ub/ghg_forcing_1955-2005_CMIP5_EnsMean.c140414.nc. Two ensemble simulations (E_2_–E_3_) use slightly different initial conditions.
E_4_ E_5_ E_6_	All forcings and design are as E_1_, except that the specified ozone forcing in the region 90°S–90°N, at 300–1 hPa was replaced by MERRA2 ozone data for the period 1979–2005. Note that the integration time of E_1_–E_6_ performed from 1979 to 2005 is because the MERRA2 data started in 1979 and simulation in WACCM4 is limited to 2005. Ozone outside of the region 90°S–90°N, at 300–1 hPa is the same as E_1_. Two ensemble simulations (E_5_–E_6_) use slightly different initial conditions.
E_7_	E_7_ is a historical simulation covering the period 1955–2005. All forcings and design are as E_1_, except that integration time from 1955 to 2005 and specified ozone forcing also from 1955 to 2005.
E_8_	All forcings and design are as E_7_. Only that the linear trend of specified ozone forcing in the region 90°S–90°N, at 300–1 hPa for the period 1955–2005 was removed. Ozone outside of the region 90°S–90°N, at 300–1 hPa is the same as E_1_.

## Simulations and Data

We used the National Center for Atmospheric Research’s CESM, version 1.0.6, which is a fully coupled global climate model that incorporates an interactive atmosphere (CAM/WACCM) component, ocean (POP2), land (CLM4), and sea ice (CICE). For the atmospheric component, we used version 4 of WACCM^65^. WACCM4 is a climate model that has detailed middle-atmosphere chemistry and a finite volume dynamical core, and it extends from the surface to approximately 140 km. For our study, we disabled the interactive chemistry (WACCM-GHG scheme). WACCM4 has 66 vertical levels, with a vertical resolution of about 1 km in the tropical tropopause and lower stratosphere layers. Simulations used a horizontal resolution of 1.9° × 2.5° (latitude × longitude) for the atmosphere and approximately the same for the ocean.

Eight transient experiments (E_1_–E_8_) with the fully coupled ocean incorporated both natural and anthropogenic external forcings, including spectrally resolved solar variability^66^, transient greenhouse gases (GHGs) (from scenario A1B of IPCC 2001), volcanic aerosols (from the Stratospheric Processes and their Role in Climate (SPARC) Chemistry–Climate Model Validation (CCMVal) REF-B2 scenario recommendations), a nudged quasi-biennial oscillation (QBO) (the time series in CESM is determined from the observed climatology over the period 1955–2005), and specified ozone forcing derived from the CMIP5 ensemble mean ozone output. An overview of all coupled experiments (E_1_–E_8_) is given in Table 1. All the forcing data used in this study are available from the CESM model input data repository.

Ozone values were obtained from the Stratospheric Water and OzOne Satellite Homogenized (SWOOSH) dataset, which is a merged record of stratospheric ozone and water vapor measurements taken by a number of limb sounding and solar occultation satellites over the previous 30 years, spanning 1984 to 2013^67^. Moreover, its primary product is a monthly-mean zonal-mean gridded dataset containing ozone and water vapor data from the SAGE-II/III, UARS HALOE, UARS MLS, and Aura MLS instruments. The horizontal resolution and vertical pressure range of the ozone data are 2.5° zonal mean (latitude: 89°S to 89°N) and 316–1 hPa (31 levels), respectively. Another ozone dataset is available from the Global Ozone Chemistry and Related trace gas Data Records for the Stratosphere (GOZCARDS, 1984–2013) project^68^. The zonal mean satellite–based GOZCARDS (1984–2013) is produced from high quality data from past missions (e.g., SAGE, HALOE data) as well as ongoing missions (ACE-FTS and Aura MLS). Its meridional resolution is 10° with 25 pressure levels from the surface up to 0.1 hPa. MERRA2 ozone (longitude × latitude resolution: 0.5° × 0.5°) uses 72 pressure levels from the surface up to 0.1 hPa^69^. The vertical resolution of MERRA2 is ~1–2 km in the upper troposphere and lower stratosphere (UTLS) and 2–4 km in the middle and upper stratosphere. MERRA2 is produced using the Goddard Earth Observing System Model, Version 5 (GEOS-5) with ozone from the Solar Backscattered Ultra Violet (SBUV) radiometers from October 1978 to October 2004, and thereafter from the Ozone Monitoring Instrument (OMI) and AURA Microwave Limb Sounder (MLS)^70^ (Bosilovich *et al*., 2015). The MERRA2 reanalysis ozone compares well with satellite ozone observations^71^ and represents the QBO and stratospheric ozone better than MERRA1^72^.

Global surface temperature data is from the NASA Goddard Institute for Space Studies (GISS) Surface Temperature Analysis (GISTEMP)^73^. GISTEMP combines land surface air temperatures primarily from the GHCN-M version 3 with sea surface temperature (SST) data from the ERSSTv3b analysis into a comprehensive global surface temperature data set spanning the period from 1880 to the present at monthly resolution, on a 2° × 2° latitude–longitude grid. The other surface temperature dataset used in this paper is version 4 of HadCRUT4, which is a set of reanalysis monthly data at a horizontal resolution of 5° × 5° from the UK Met Office Hadley Centre and the University of East Anglia’s Climatic Research Unit^74^ that combines the global land surface temperature data set, CRUTEM4, and the global SST data set, HadSST3. SST data were obtained from the UK Met Office Hadley Centre for Climate Prediction and Research SST (HadSST) field datasets. Geopotential height, temperature, and zonal wind were obtained from the ERA-Interim reanalysis.

## Electronic supplementary material

Supplementary Figure S1-5
