# Supplementary material for: Improved Global Surface Temperature Simulation using Stratospheric Ozone Forcing with More Accurate Variability
Source: Sci Rep. 2018 Sep 27;8:14474. doi: 10.1038/s41598-018-32656-z (PMC6160484; doi:10.1038/s41598-018-32656-z)
Supplement: Supplementary file 1 — Supplementary Figure S1-5 [file 41598_2018_32656_MOESM1_ESM.docx]

**Supplementary Information of paper:**

**Improved Global Surface Temperature Simulation using Stratospheric Ozone Forcing with More Accurate Variability**

Fei Xie^1^, Jianping Li^1,2*^, Cheng Sun^1^, Ruiqiang Ding^3^, Nan Xing^4^,

Yun Yang^1^, Xin Zhou^5^, Xuan Ma^1^

*^1^College of Global Change and Earth System Science, Beijing Normal University, Beijing, China*

*^2^Laboratory for Regional Oceanography and Numerical Modeling, Qingdao National Laboratory for Marine Science and Technology, Qingdao, China*

*^3^State Key Laboratory of Numerical Modeling for Atmospheric Sciences and Geophysical Fluid Dynamics, Institute of Atmospheric Physics, Chinese Academy of Sciences, Beijing, China*

*^4^Beijing Meteorological Observatory, Beijing, China*

*^5^Plateau Atmosphere and Environment Key Laboratory of Sichuan Province, College of Atmospheric Science , Chengdu University of Information Technology, Chengdu, China*


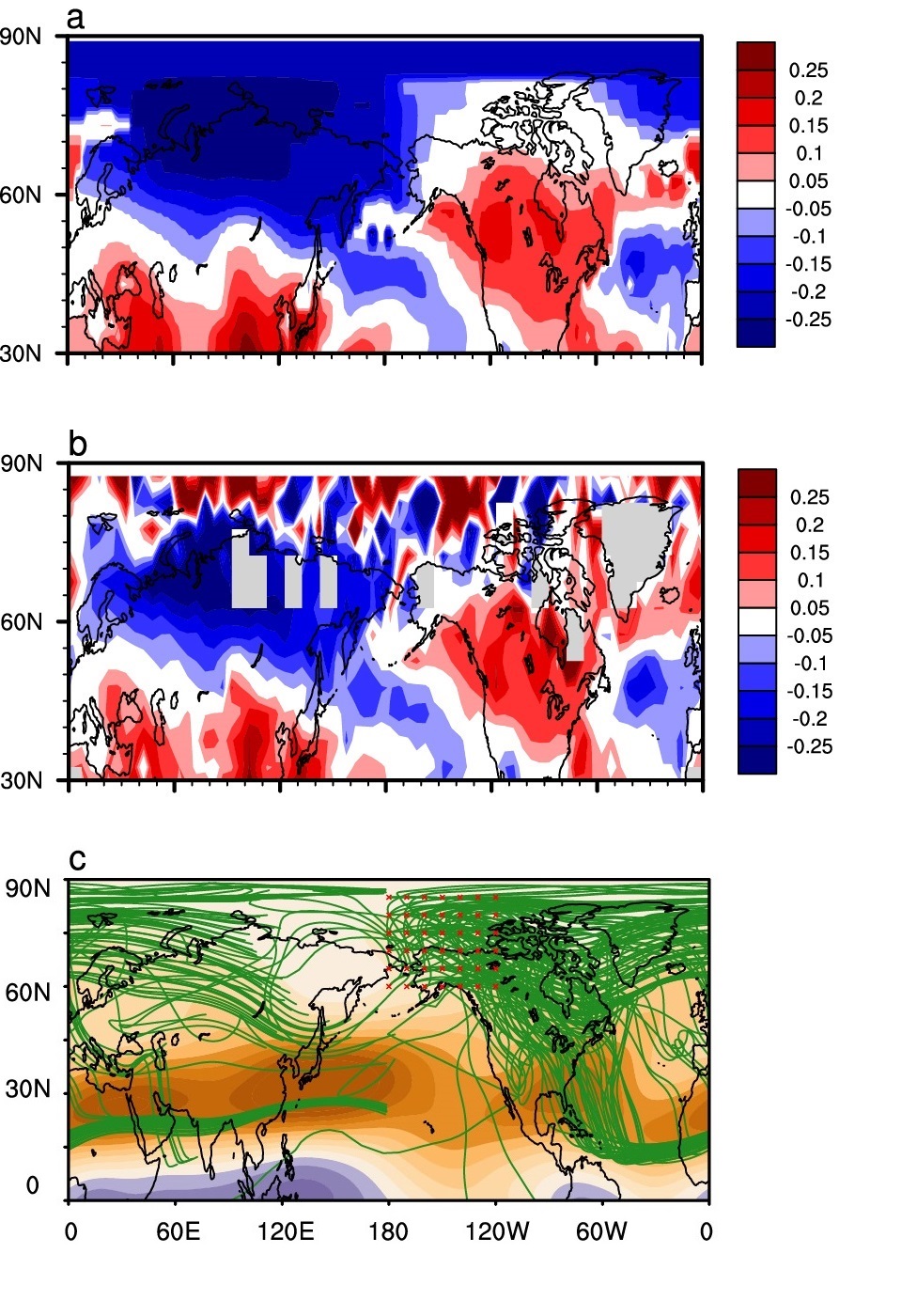


**Figure S1 |** (a) Horizontal distribution of correlation coefficients between surface temperature variations from GISTEMP and stratospheric ozone averaged over the region 60–90°N and 150–50 hPa from SWOOSH for the period 1979–2005. (b) Same as (a), but for surface temperature variations from HadCRUT4. The variations have the seasonal cycle removed and are detrended.


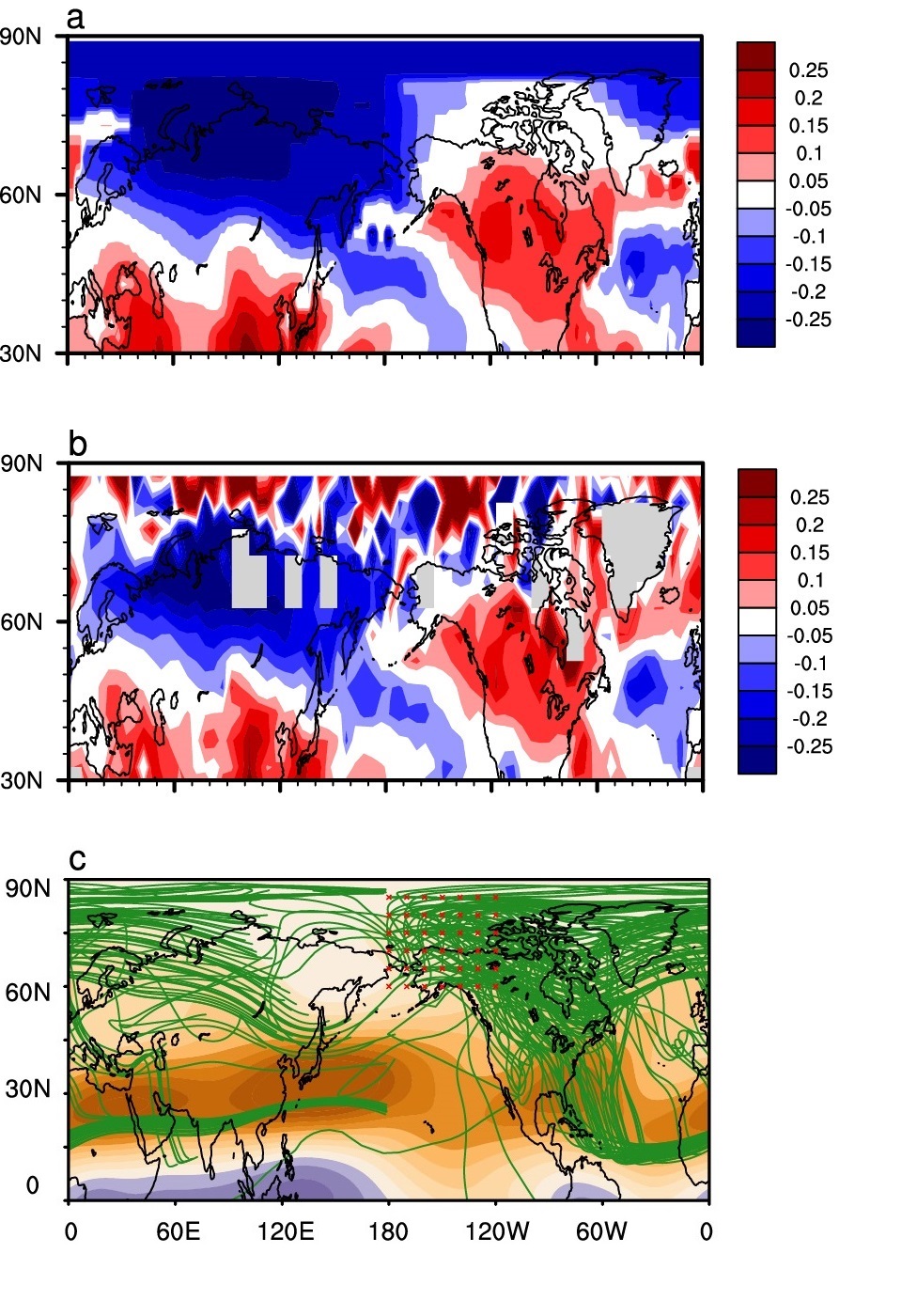


**Figure S2 |** Ray paths (green lines) at 200 hPa after the circulation was perturbed for 15 days. Red dots denote wave sources in the region 60°–90°N, 180°–120°W at 200 hPa. The wavenumbers along these rays are in the range 1–3. Color shading indicates the climatological flow.


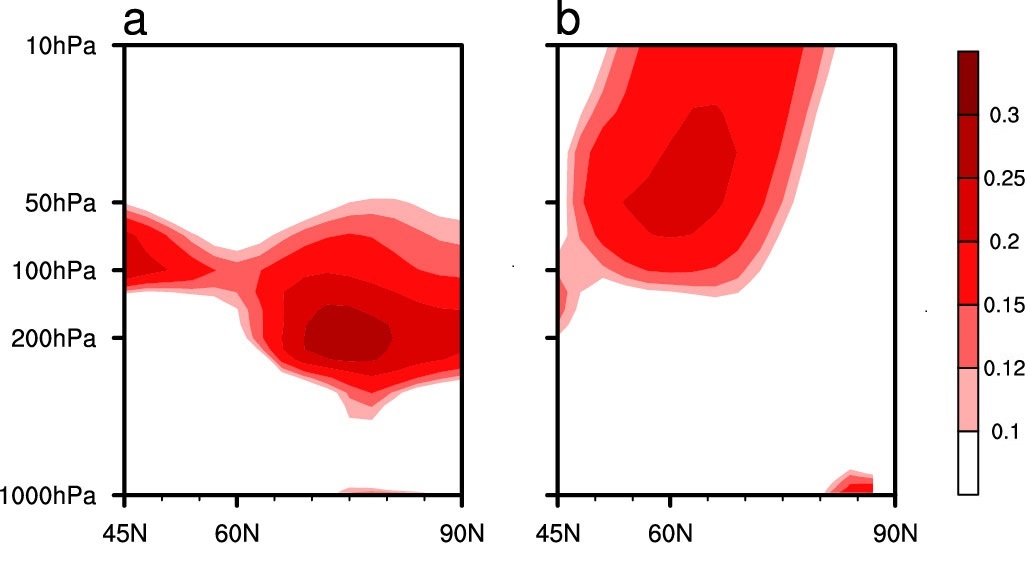


**Figure S3 |** Difference between the correlation coefficients for stratospheric temperature from E_4-6_ against ERA-Interim and the correlation coefficients for stratospheric temperature from E_1–3_ against ERA-Interim for the period 1979–2005 in the Northern Hemisphere. (b) Same as (a), but for stratospheric circulation (U).


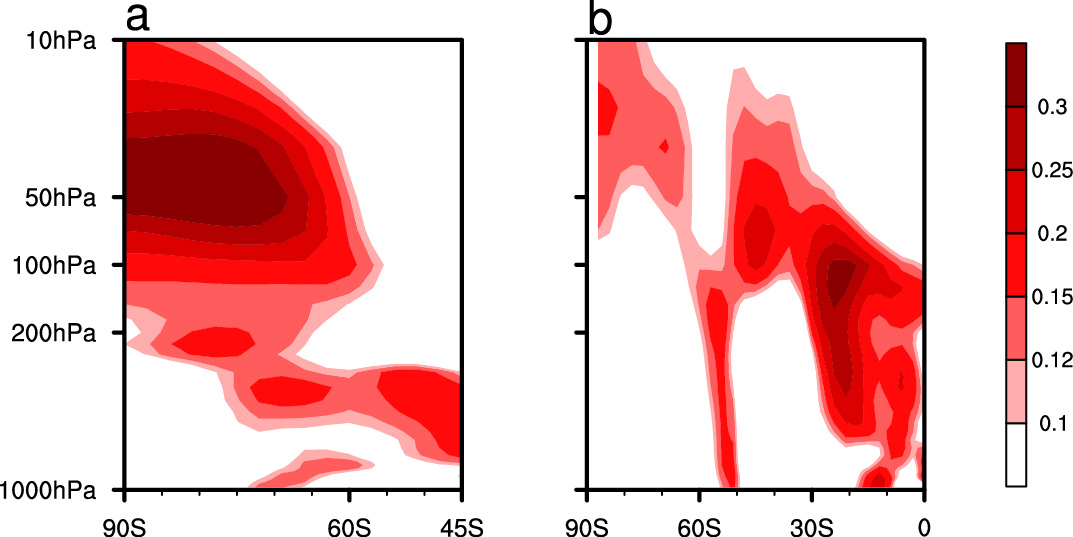


**Figure S4 |** (a) and (b) are same as Fig. S3a and b, but in the Southern Hemisphere.


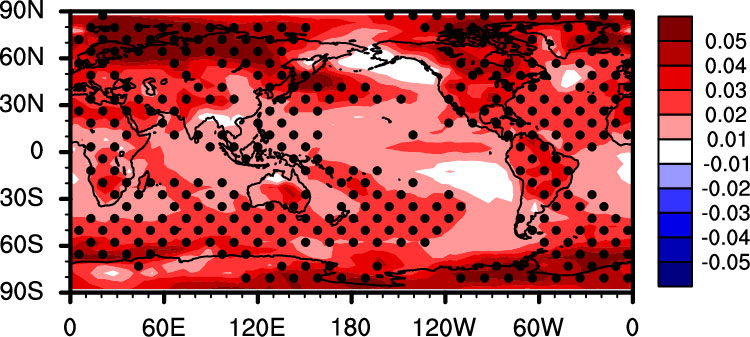


**Figure S5 |** Same as Fig. 5b, but for the period 1955–1995.
